# The diagnostic performance of quantitative flow ratio and perfusion imaging in patients with prior coronary artery disease

**DOI:** 10.1093/ehjci/jead197

**Published:** 2023-08-14

**Authors:** Pepijn A van Diemen, Ruben W de Winter, Stefan P Schumacher, Henk Everaars, Michiel J Bom, Ruurt A Jukema, Yvemarie B Somsen, Pieter G Raijmakers, Rolf A Kooistra, Janny Timmer, Teemu Maaniitty, Lourens F Robbers, Martin B von Bartheld, Ahmet Demirkiran, Albert C van Rossum, Johan H Reiber, Juhani Knuuti, S Richard Underwood, Eike Nagel, Paul Knaapen, Roel S Driessen, Ibrahim Danad

**Affiliations:** Department of Cardiology, Amsterdam UMC, Vrije Universiteit Amsterdam, De Boelelaan 1117, Amsterdam 1081 HV, The Netherlands; Department of Cardiology, Amsterdam UMC, Vrije Universiteit Amsterdam, De Boelelaan 1117, Amsterdam 1081 HV, The Netherlands; Department of Cardiology, Amsterdam UMC, Vrije Universiteit Amsterdam, De Boelelaan 1117, Amsterdam 1081 HV, The Netherlands; Department of Cardiology, Amsterdam UMC, Vrije Universiteit Amsterdam, De Boelelaan 1117, Amsterdam 1081 HV, The Netherlands; Department of Cardiology, Amsterdam UMC, Vrije Universiteit Amsterdam, De Boelelaan 1117, Amsterdam 1081 HV, The Netherlands; Department of Cardiology, Amsterdam UMC, Vrije Universiteit Amsterdam, De Boelelaan 1117, Amsterdam 1081 HV, The Netherlands; Department of Cardiology, Amsterdam UMC, Vrije Universiteit Amsterdam, De Boelelaan 1117, Amsterdam 1081 HV, The Netherlands; Department of Radiology and Nuclear Medicine, Amsterdam UMC, Vrije Universiteit Amsterdam, Amsterdam, The Netherlands; Medis Medical Imaging, Leiden, The Netherlands; Medis Medical Imaging, Leiden, The Netherlands; Turku PET Centre, Turku University Hospital and University of Turku, Turku, Finland; Department of Cardiology, Amsterdam UMC, Vrije Universiteit Amsterdam, De Boelelaan 1117, Amsterdam 1081 HV, The Netherlands; Department of Cardiology, Amsterdam UMC, Vrije Universiteit Amsterdam, De Boelelaan 1117, Amsterdam 1081 HV, The Netherlands; Department of Cardiology, Amsterdam UMC, Vrije Universiteit Amsterdam, De Boelelaan 1117, Amsterdam 1081 HV, The Netherlands; Department of Cardiology, Amsterdam UMC, Vrije Universiteit Amsterdam, De Boelelaan 1117, Amsterdam 1081 HV, The Netherlands; Medis Medical Imaging, Leiden, The Netherlands; Turku PET Centre, Turku University Hospital and University of Turku, Turku, Finland; Department of Nuclear Medicine, Royal Brompton Hospital, London, UK; Institute of Experimental and Translational Cardiovascular Imaging, DZHK Centre for Cardiovascular Imaging, University Hospital Frankfurt am Main, Frankfurt am Main, Germany; Department of Cardiology, Amsterdam UMC, Vrije Universiteit Amsterdam, De Boelelaan 1117, Amsterdam 1081 HV, The Netherlands; Department of Cardiology, Amsterdam UMC, Vrije Universiteit Amsterdam, De Boelelaan 1117, Amsterdam 1081 HV, The Netherlands; Department of Cardiology, Amsterdam UMC, Vrije Universiteit Amsterdam, De Boelelaan 1117, Amsterdam 1081 HV, The Netherlands

**Keywords:** fractional flow reserve, single-photon emission computed tomography, positron emission tomography, cardiac magnetic resonance imaging, quantitative flow ratio, chronic coronary syndrome

## Abstract

**Aims:**

In chronic coronary syndrome (CCS) patients with documented coronary artery disease (CAD), ischaemia detection by myocardial perfusion imaging (MPI) and an invasive approach are viable diagnostic strategies. We compared the diagnostic performance of quantitative flow ratio (QFR) with single-photon emission computed tomography (SPECT), positron emission tomography (PET), and cardiac magnetic resonance imaging (CMR) in patients with prior CAD [previous percutaneous coronary intervention (PCI) and/or myocardial infarction (MI)].

**Methods and results:**

This PACIFIC-2 sub-study evaluated 189 CCS patients with prior CAD for inclusion. Patients underwent SPECT, PET, and CMR followed by invasive coronary angiography with fractional flow reserve (FFR) measurements of all major coronary arteries (*N* = 567), except for vessels with a sub-total or chronic total occlusion. Quantitative flow ratio computation was attempted in 488 (86%) vessels with measured FFR available (FFR ≤0.80 defined haemodynamically significant CAD). Quantitative flow ratio analysis was successful in 334 (68%) vessels among 166 patients and demonstrated a higher accuracy (84%) and sensitivity (72%) compared with SPECT (66%, *P* < 0.001 and 46%, *P* = 0.001), PET (65%, *P* < 0.001 and 58%, *P* = 0.032), and CMR (72%, *P* < 0.001 and 33%, *P* < 0.001). The specificity of QFR (87%) was similar to that of CMR (83%, *P* = 0.123) but higher than that of SPECT (71%, *P* < 0.001) and PET (67%, *P* < 0.001). Lastly, QFR exhibited a higher area under the receiver operating characteristic curve (0.89) than SPECT (0.57, *P* < 0.001), PET (0.66, *P* < 0.001), and CMR (0.60, *P* < 0.001).

**Conclusion:**

QFR correlated better with FFR in patients with prior CAD than MPI, as reflected in the higher diagnostic performance measures for detecting FFR-defined, vessel-specific, significant CAD.

## Graphical Abstract

**Graphical Abstract:**
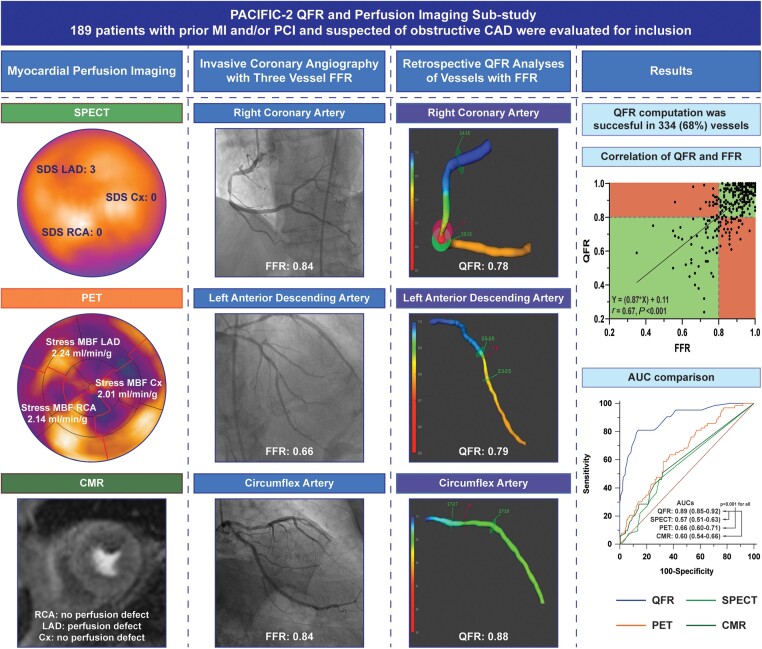
This sub-study of the PACIFIC-2 compared the diagnostic performance of QFR with that of SPECT, PET, and CMR when referenced by FFR. A stress MBF ≤2.3 mL/min/g in two adjacent segments on PET, an SDS ≥1 on SPECT, a visually assessed perfusion defect on CMR, and QFR ≤0.80 were used to define the presence of ischaemia. Retrospective QFR analyses were attempted in all vessels with FFR and were successful in 334 (68%) vessels. In comparative AUC analyses, QFR exhibited a higher AUC than SPECT, PET, and CMR. Note: SPECT, PET, CMR, invasive angiography, and QFR images are derived from a single patient. MBF, myocardial blood flow; PCI, percutaneous coronary intervention; SDS, summed difference score; other abbreviations as in *Figures 1*, *3*, and *4*.

## Introduction

In patients with coronary artery disease (CAD), fractional flow reserve (FFR)-guided percutaneous coronary intervention (PCI) leads to a greater reduction in symptom burden and lower event rate as compared with medical therapy (MT) alone.^1,2^ An array of diagnostic techniques are available to select patients who may benefit from potential revascularization.^3^ For patients with a high likelihood of obstructive CAD, guidelines support non-invasive ischaemia testing or a direct invasive approach.^3,4^ Myocardial perfusion imaging (MPI) can be utilized when a non-invasive strategy is desired.^3^ If an invasive approach is preferred, haemodynamic significance of epicardial disease should be determined by means of pressure wire-derived measures (e.g. FFR), given the discrepancy between visually determined severity of CAD and the actual functional repercussion.^3–5^ Utilization of these techniques is limited to intervention centres, and despite the beneficial outcome of FFR-guided revascularization, its implementation is not routine clinical practice because of the additional costs and risks, need to induce hyperaemia, prolonged procedure time, and operator confidence that angiographic assessment alone suffices.^6,7^ Quantitative flow ratio (QFR) may overcome some of these limitations as it uses fast fluid dynamics and a 3D reconstruction of the coronary artery derived from invasive coronary angiography (ICA) to model FFR, obviating the need to use pressure wires and induce hyperaemia.^8^ This study compared the diagnostic performance of QFR with MPI by single-photon emission computed tomography (SPECT), positron emission tomography (PET), and cardiac magnetic resonance imaging (CMR) in patients with prior myocardial infarction (MI) and/or PCI.

## Methods

### Study design and population

This is a sub-study of the PACIFIC-2 study, which included 189 symptomatic chronic coronary syndrome (CCS) patients with prior MI and/or PCI who were referred for ICA.^9^ Patients underwent SPECT, PET, and CMR prior to ICA. During ICA, all coronary arteries were interrogated by FFR regardless of stenosis severity and imaging results. In- and exclusion criteria have previously been described.^9^ Retrospective QFR computation was attempted in vascular territories in which FFR was obtained. Vascular territories with a sub-total or total occlusion and no FFR ≤ 0.80 were excluded. Vascular territories with a sub-total or total occlusion and also an FFR ≤0.80 were included. Vessels with angiographic or lesion characteristics prohibiting QFR analyses were excluded (see Supplementary data online, *S1*). The PACIFIC-2 was approved by the institutional Medical Ethics Committee and complied with the Declaration of Helsinki. All patients provided written informed consent. The data underlying this article will be shared on reasonable request to the corresponding author.

### Single-photon-emission computed tomography

SPECT acquisition and assessment were performed as previously described.^9^ Scans were obtained on a SPECT/CT scanner (Symbia T2, Siemens, Erlanger, Germany) using a stress (intravenous adenosine 140 µg/kg/min) and rest protocol using ^99^mTc tetrofosmin. A blinded core laboratory (Royal Brompton Hospital, London, UK) analysed images using a 17-segment model wherein every segment was graded for the presence of a perfusion defect on a 5-point scale.^10^ A summed difference score (SDS) ≥1 within a vascular territory was indicative of myocardial ischaemia. Vascular perfusion defect percentage was calculated as: (SDS/maximal achievable SDS) × 100.

### Positron emission tomography

PET methodology has previously been described.^9^ Images were acquired on a PET/CT device (Ingenuity, Philips Healthcare, Best, The Netherlands) using a rest and stress (intravenous adenosine 140 µg/kg/min) protocol and 370 MBq of [^15^O]H_2_O. Images were assessed by a blinded core laboratory (Turku University Hospital, Turku, Finland). Absolute hyperaemic myocardial blood flow (MBF) ≤2.3 mL/min/g in ≥2 adjacent segments within a vascular territory was considered indicative of ischaemia.^10,11^ Vascular hyperaemic MBF was defined as the hyperaemic MBF of the vascular territory in the absence of a perfusion defect or as the hyperaemic MBF of the perfusion defect (≥2 adjacent segments with a hyperaemic MBF ≤2.3 mL/min/g) when present.

### Cardiac magnetic resonance imaging

CMR acquisition and assessment were performed as previously described.^9^ CMR was obtained on a 1.5 Tesla scanner (Magnetom Avanto, Siemens). Perfusion images were acquired using three parallel short-axis at the basal, mid, and apical levels and obtained every heartbeat for 50–70 cardiac cycles following the injection of a gadolinium-based contrast agent during hyperaemia (intravenous adenosine 140 µg/kg/min). Late gadolinium enhancement (LGE) was performed using a 2D-segmented inversion-recovery gradient-echo pulse sequence. A blinded core laboratory under supervision of the Institute of Experimental and Translational Cardiovascular Imaging (University Hospital Frankfurt am Main, Frankfurt am Main, Germany) visually assessed LGE and perfusion images using a 17-segment model excluding the apex.^10^ LGE and perfusion defect per segment were scored using a five-point scale according to amount of LGE and perfusion defect (0, 1–25, 26–50, 51–75, and >75%). The presence of ischaemia was defined as a perfusion defect within a vascular territory extending beyond LGE, or in the absence of LGE as: a perfusion defect circumferential of >1 segment, a perfusion defect extending >1 slice, or a perfusion defect with >50% transmurality. A vascular LGE score >1 was used to define the presence of MI. Segmental perfusion defect scores were calculated by subtracting the LGE score from the perfusion defect score (with a minimum score of 0). Vascular perfusion defect percentage was defined as (perfusion defect score/maximal achievable perfusion defect score) × 100.

### Quantitative flow ratio

QFR® analyses were retrospectively performed by a blinded core laboratory (ClinFact Medis, Leiden, The Netherlands) using the QAngio XA 3D/QFR® V2.0 software package (Medis Medical Imaging Systems, Leiden, The Netherlands). Two end-diastolic frames at least 25° apart from the coronary of interest were used to reconstruct a 3D model of the coronary. The reference diameter of the vessel was constructed by marking healthy coronary segments preferably proximal and distal to a lesion of interest. Intermediate lesions were defined as a 3D quantitative coronary angiography diameter stenosis from 30 to 90%. Contrast frame counting was performed to obtain an estimated contrast flow velocity, which is converted into a virtual hyperaemic flow velocity. For all vessels, contrast QFR was computed based on the performed frame counting, and fixed QFR was computed based on an empiric hyperaemic flow velocity.^8^ Contrast QFR was used to define the QFR value of the vessel except for 17 vessels in which frame counting could not be performed. QFR ≤0.80 defined significant CAD. The QFR analysis time was around 3 min per vessel.

### ICA and FFR

Vasodilation was achieved by an intracoronary injection of nitroglycerine. Images were obtained in at least two projections per evaluated coronary artery. In the PACIFIC-2 study, 80 patients (240 vascular territories) underwent ICA without adherence to a QFR acquisition protocol, and in 109 patients (327 vascular territories), a standardized QFR acquisition protocol was utilized in which recommended QFR angles were obtained (see Supplementary data online, *Table S1*). Furthermore, images were shot at a frame rate of at least 12.5 frames/s and without the use of magnification and/or panning. After visualization, major coronary arteries were interrogated by FFR regardless of stenosis severity except for vessels with a sub-total/total occlusion or severe tortuosity in which wire passage was not deemed feasible. FFR ≤0.80 defined significant CAD, and FFR ≥0.75 and ≤0.85 defined a grey-zone FFR.^12,13^

### Statistical analysis

Analyses were performed using SPSS (IBM SPSS Statistics 26.0, Armonk, NY, USA) and MedCalc (MedCalc Software 12.7.8.0, Mariakerke, Belgium). Normally distributed variables are presented as mean ± SD and non-normally distributed continuous variables as median (inter-quartile range). Categorical variables are shown as frequencies with percentages. Per vessel diagnostic performance measures with 95% confidence intervals were calculated and compared using generalized estimating equations that accounted for multiple measurements (vessels and diagnostic modalities) within a patient using an exchangeable correlation structure (sensitivity, specificity, and accuracy) or an independent correlation structure [negative predictive value (NPV) and positive predictive value (PPV)]. Area under the receiver operating characteristic curves were constructed using QFR, vascular hyperaemic MBF (PET), and vascular perfusion defect % for SPECT and CMR and compared using the DeLong method. Correlation of QFR and FFR was quantified using Spearman’s correlation coefficients, and agreement was visualized with Bland–Altman plots and quantified with intra-class correlation coefficients (ICCs). A two-way mixed effects model was used to determine the ICC for single measures. Bias between QFR and FFR was assessed using paired Student’s *t*-tests. Differences in QFR analysis success rate were assessed with a Fisher’s exact test. A two-sided *P*-value <0.05 was considered statistically significant.

## Results

### Study population

**Figure 1:**
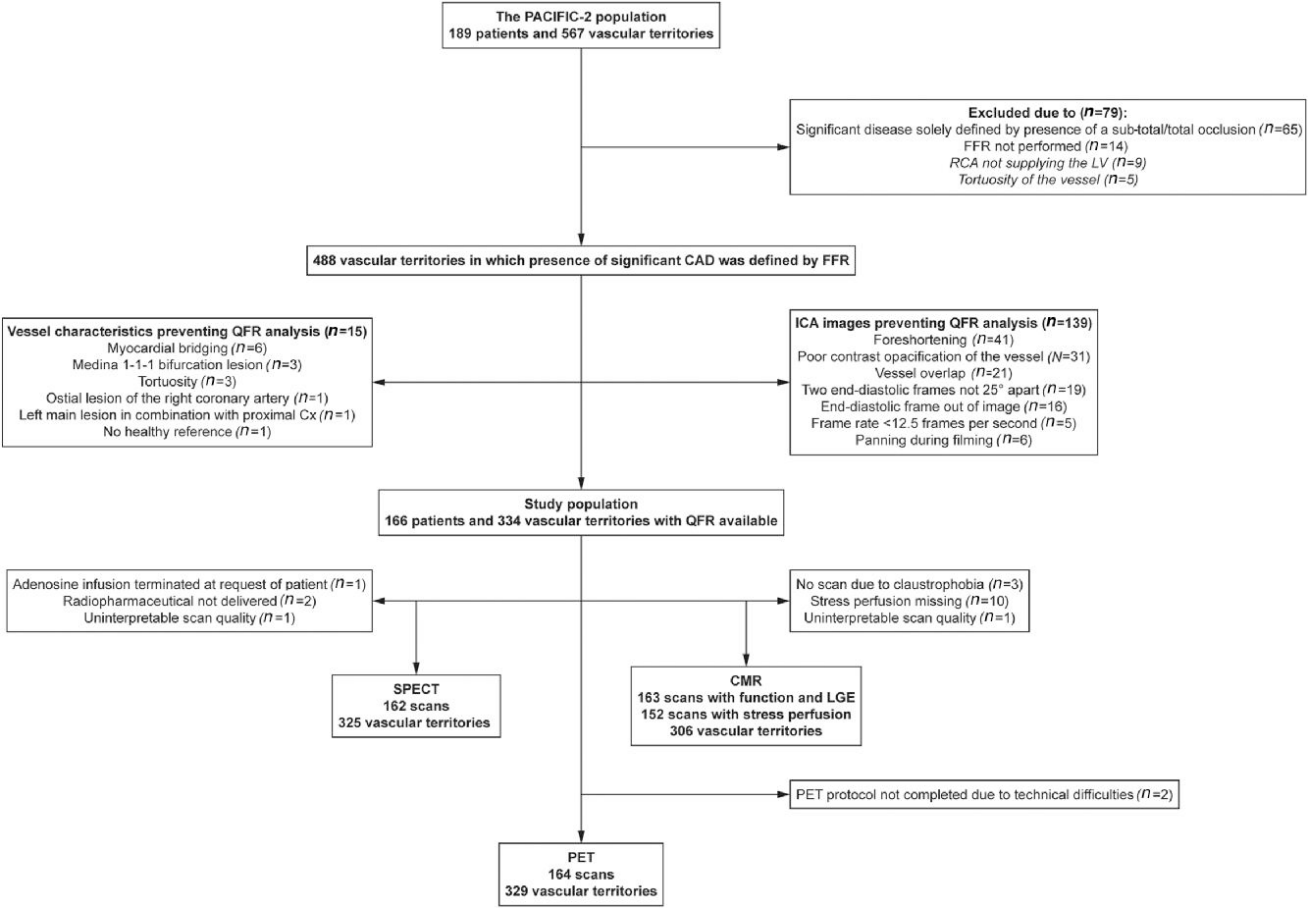
Flowchart of in- and excluded vessels. Cx, circumflex artery; LV, left ventricle; RCA, right coronary artery.

Of the 567 vascular territories evaluated, 14 (2%) were excluded because of the absence of FFR and 65 (11%), because significant disease was solely defined by a sub-total/total occlusion (*Figure 1*). Among the remaining vessels, 15 (3%) were excluded because of vessel or lesions characteristics prohibiting QFR analysis, and an additional 139 (28%) were excluded because of inadequate ICA images. This resulted in 166 patients (78% male, age: 63.1 ± 9.3 years) with at least one successful QFR analysis. Patient, angiographic, physiological, and imaging characteristics are presented in *Tables 1* and *2*, respectively (Supplementary data online, *Tables S2* and *S3* display characteristics of in- and excluded patients/vessels). Among the 334 vascular territories with a QFR result, assessment of ischaemia by means of SPECT, PET, or CMR was available in 325, 329, and 306 territories, respectively (*Figure 1*).

**Table 1:** Patient characteristics

Patient characteristics	*N* = 166
Male gender	130 (78)
Age in years	63.1 ± 9.3
Body mass index (kg/m^2^)	27.3 ± 4.2
Cardiovascular risk factors	
Diabetes mellitus	35 (21)
Hypertension	105 (63)
Hypercholesterolaemia	113 (68)
Current smoker	22 (13)
History of smoking	71 (43)
Family history of CAD	86 (52)
Medication	
Single antiplatelet therapy	106 (64)
Dual antiplatelet therapy	59 (36)
Beta-blocker	102 (61)
Calcium channel blocker	59 (36)
Statin	142 (86)
Long acting nitrate	38 (23)
ACE-inhibitor or AR-blocker	95 (57)
Cardiac history	
Previous PCI	157 (93)
Previous MI	87 (52)
Symptoms	
Typical angina pectoris	64 (39)
Atypical angina pectoris	41 (25)
Non-specific chest pain	25 (15)
Dyspnoea	36 (22)
Left ventricular function	
LVEF%^a^	58.7 ± 8.5
≥55%	124 (75)
45 to <55%	30 (18)
35 to <45%	9 (5)
≤35%	3 (2)
Invasive coronary angiography	
Significant CAD	100 (60)

Values are presented as mean ± SD, median (inter-quartile range), or absolute numbers (%).

ACE, angiotensin-converting enzyme; AR, angiotensin receptor; LVEF, left ventricular ejection fraction.

^a^LVEF as measured on CMR. SPECT resting LVEF was used for three patients without CMR.

**Table 2:** Angiographic, physiological, and imaging characteristics of the included vessels

Vascular territory	*n* = 334
Right coronary artery	93 (28)
Left anterior descending artery	127 (38)
Circumflex artery	114 (34)
Anatomical lesion characteristics	
Lesion length (mm)	16.3 (10.5–26.2)
Diameter stenosis (%)	41 ± 14
Intermediate lesions	258 (77)
Area stenosis (%)	56 ± 18
Minimal lumen diameter (mm)	1.7 ± 0.5
Invasive physiology characteristics	
QFR	0.92 (0.80–0.98)
QFR ≤0.80	85 (25)
FFR	0.90 (0.82–0.96)
FFR ≤0.80	72 (22)
Grey zone FFR (≥0.75 and ≤0.85)	78 (23)
PET (*n* = 329)	
Hyperaemic MBF (mL/min/g)	2.79 ± 0.98
Indicative of ischaemia	127 (38)
SPECT (*n* = 325)	
SDS	0 (0–1)
Perfusion defect percentage	0 (0–5)
Normal scan	162 (51)
Fixed perfusion defect	57 (18)
Reversible perfusion defect	55 (17)
Mixed perfusion defect	51 (16)
Indicative of ischaemia	106 (33)
CMR (LGE *n* = 328 and perfusion *n* = 306)	
LGE score	0 (0–1)
MI	76 (23)
Perfusion defect score	0 (0–3)
Perfusion defect percentage	0 (0–13)
Indicative of ischaemia	64 (21)

Values are presented as mean ± SD, median (inter-quartile range), or absolute numbers (%).

### QFR analysis success rate

QFR analyses were successful in 68% of the vessels (*Figure 1*). QFR success rate was higher among vessels that were acquired using the QFR protocol compared with those obtained without (81 vs. 52%, *P* < 0.001). The higher success rate was driven by a reduction in the number of vessels with ICA images unsuitable for QFR analyses (48 vs. 15%, *P* < 0.001) (see Supplementary data online, *Table S4*).

### QFR vs. FFR

**Figure 2:**
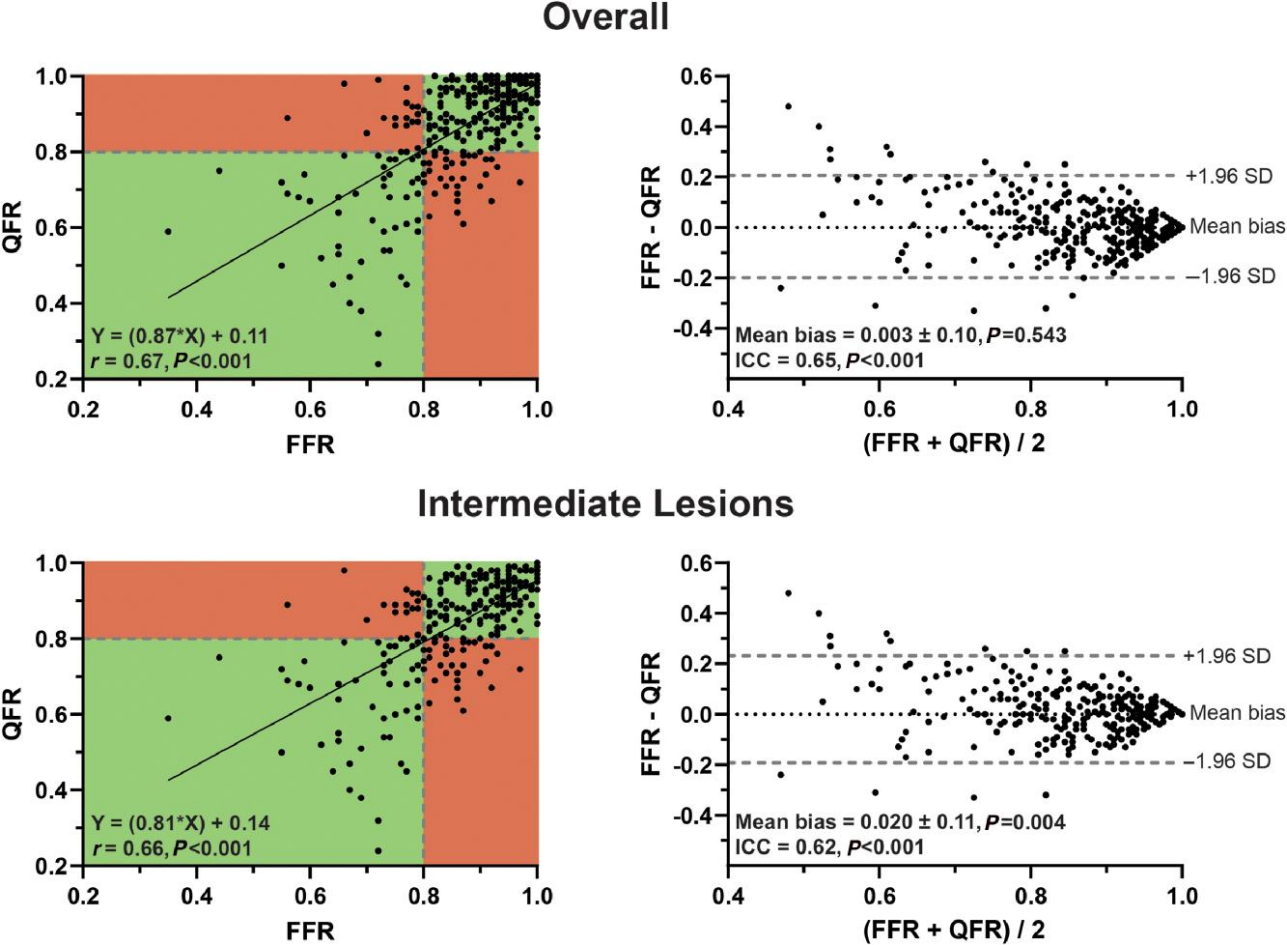
Correlation and agreement of QFR and FFR. Scatterplots with Spearman’s correlation coefficient and linear regression equations and Bland–Altman plots with ICCs.

**Figure 3:**
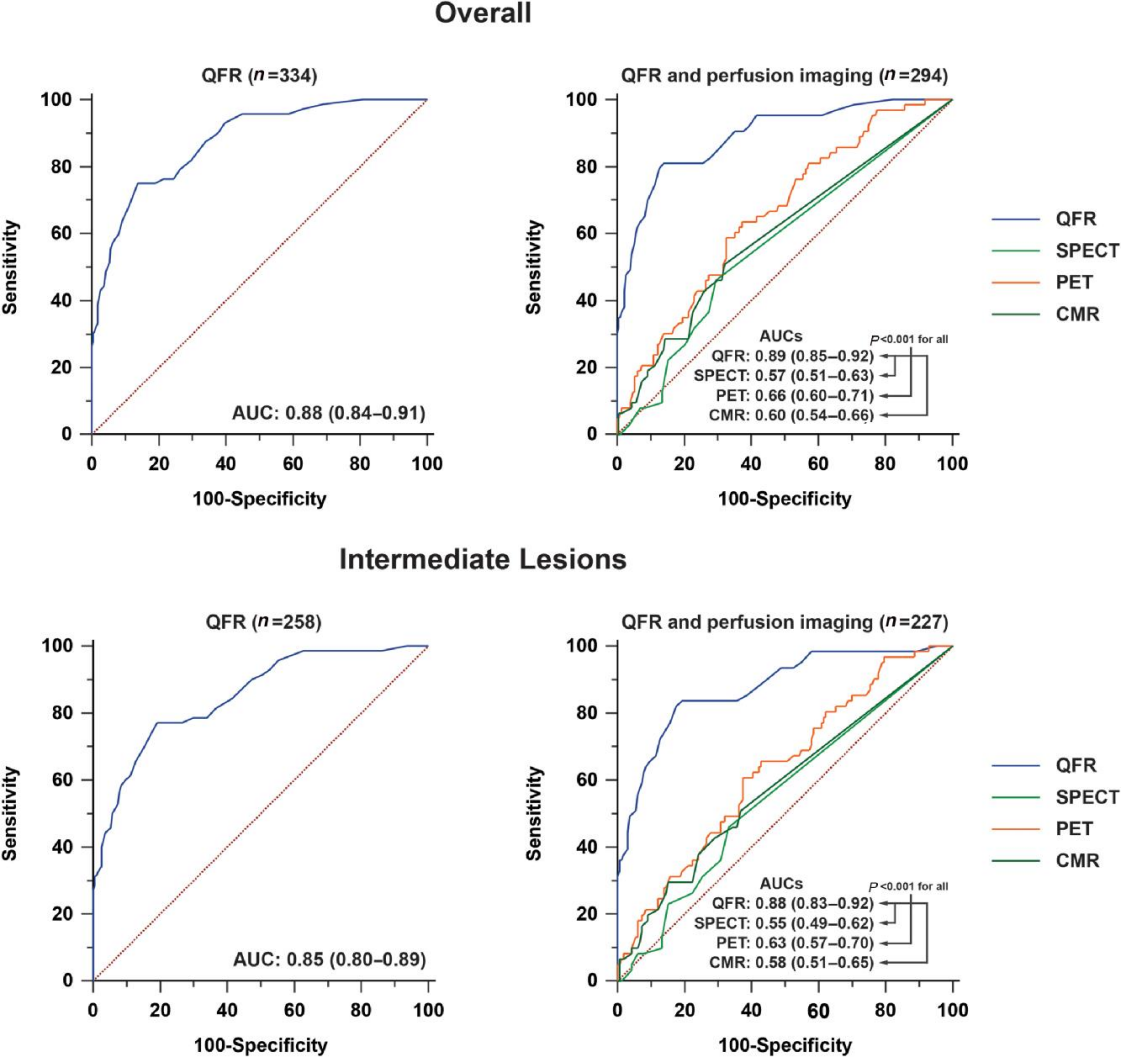
AUCs of QFR and perfusion imaging. *P*-values concern the comparison of QFR with SPECT, PET, and CMR.

**Figure 4:**
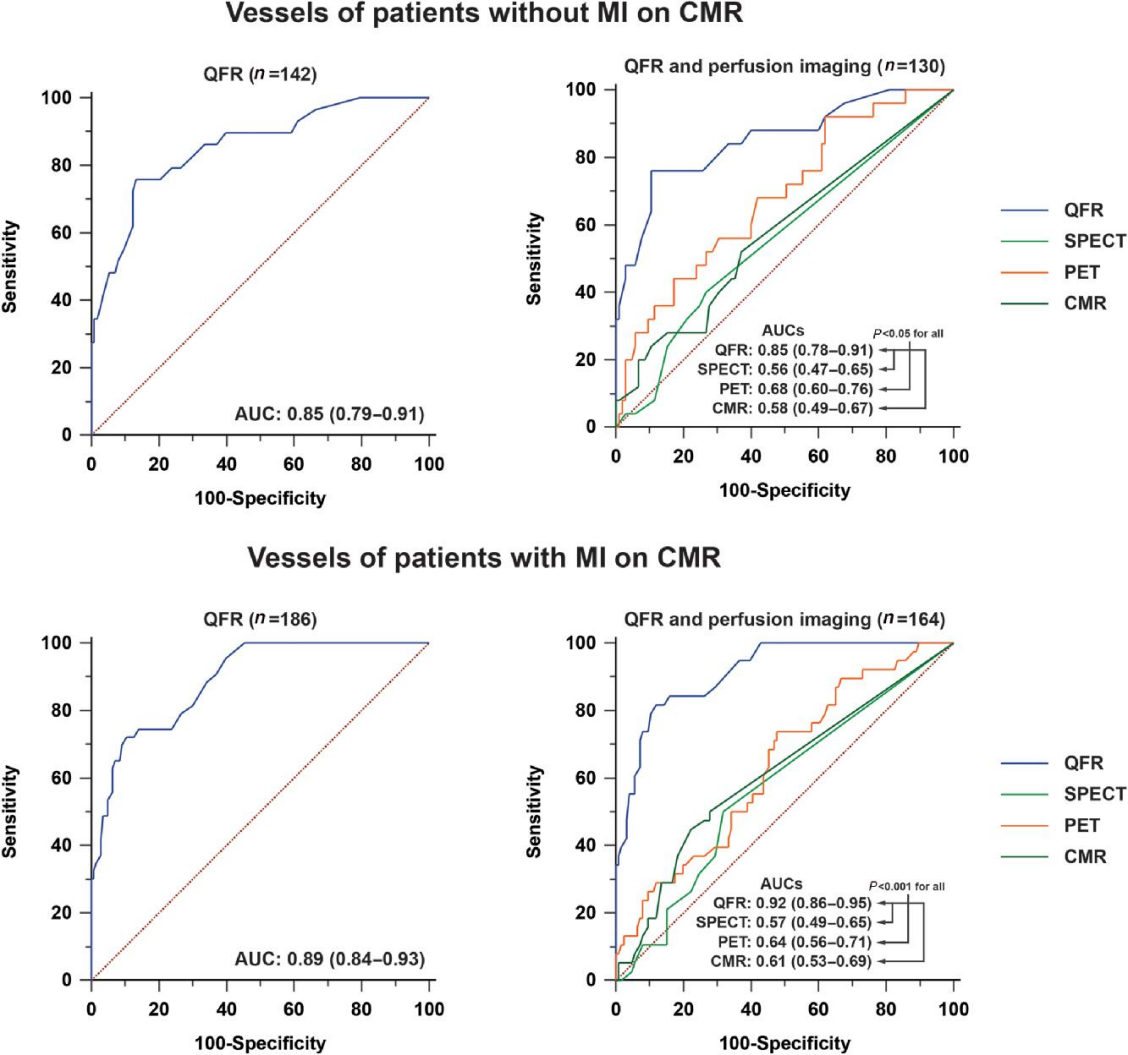
AUCs of QFR and perfusion imaging in vessels with and without MI on CMR. *P*-values concern the comparison of QFR with SPECT, PET, and CMR.

A significant correlation and agreement between QFR and FFR were observed (*Figure 2*). Overall, mean FFR and QFR did not differ, whereas mean FFR was higher than mean QFR in vessels with an intermediate lesion (FFR: 0.86 ± 0.11 vs. QFR: 0.84 ± 0.14, *P* = 0.004) (*Figure 2*). The diagnostic performance of QFR is presented in *Tables 3* and *4* and *Figures 3* and *4*. Fixed and contrast QFR correlated and were in agreement with FFR to a similar extent (fixed QFR: *r* = 0.68 and ICC = 0.66, contrast QFR: *r* = 0.67 and ICC = 0.66, *P* < 0.001 for all) (see Supplementary data online, *Figure S1*). There were no differences between the diagnostic performance measures of fixed and contrast QFR, respectively (see Supplementary data online, *Table S5* and *Figure S2*).

### Diagnostic comparison of QFR and perfusion imaging to detect FFR-defined CAD

QFR exhibited a higher sensitivity, NPV, PPV, accuracy, and AUC when compared with SPECT, PET, and CMR (*Table 3* and *Figure 3*). Specificity of QFR was similar to that of CMR and higher than SPECT and PET. In vessels of patients without MI on CMR, QFR had a higher sensitivity, NPV, PPV, accuracy, and AUC in comparison with SPECT, PET, and CMR, whereas specificity of QFR was not different from PET and CMR but was higher than SPECT (*Table 4* and *Figure 4*). In vessels of patients with MI on CMR, QFR had a superior NPV, PPV, accuracy, and AUC compared with SPECT, PET, and CMR, whereas its sensitivity was comparable with PET but higher than that of SPECT and CMR and its specificity was similar to CMR but greater than that of SPECT and PET (*Table 4* and *Figure 4*). In vessels with MI on CMR in the respective vascular territory (*N* = 76), QFR had a higher accuracy as compared with SPECT (87 vs. 57%, *P* < 0.001) and PET (53%, *P* < 0.001) while similar to CMR (76%, *P* = 0.096), and the AUC of QFR (0.96) was higher than SPECT (0.52, *P* < 0.001), PET (0.57, *P* < 0.001), and CMR (0.61, *P* < 0.001) (see Supplementary data online, *Table S6* and *Figure S3*). Supplementary data online, *Table S7* presents the diagnostic performance of QFR and MPI modalities among vessels with an FFR outside the grey zone.

**Table 3:** Per vessel diagnostic performance, overall and among vessels with a lesion of intermediate severity

Overall							
	QFR (*n* = 334)	SPECT (*n* = 325)	*P*-value	PET (*n* = 329)	*P*-value	CMR (*n* = 306)	*P*-value
Sensitivity	72 (61–81)	46 (34–57)	0.001	58 (46–69)	0.032	33 (23–45)	<0.001
Specificity	87 (83–91)	71 (65–76)	<0.001	67 (61–72)	<0.001	83 (77–87)	0.123
NPV	92 (88–95)	83 (77–87)	<0.001	86 (80–90)	0.003	82 (76–86)	<0.001
PPV	61 (50–71)	30 (22–40)	<0.001	32 (25–41)	<0.001	34 (24–47)	<0.001
Accuracy	84 (80–88)	66 (60–71)	<0.001	65 (60–70)	<0.001	72 (67–77)	<0.001

Intermediate lesions							
	QFR (*n* = 258)	SPECT (*n* = 252)	*P*-value	PET (*n* = 253)	*P*-value	CMR (*n* = 237)	*P*-value
Sensitivity	74 (63–83)	45 (34–57)	<0.001	60 (48–71)	0.033	34 (24–46)	<0.001
Specificity	82 (76–87)	67 (60–74)	0.001	62 (55–69)	<0.001	80 (74–86)	0.608
NPV	90 (84–93)	77 (70–83)	<0.001	81 (74–87)	0.004	77 (70–82)	<0.001
PPV	61 (50–71)	34 (25–44)	<0.001	37 (28–46)	<0.001	39 (27–53)	0.001
Accuracy	80 (75–85)	62 (55–67)	<0.001	62 (55–67)	<0.001	0.68 (62–73)	0.001

Values are presented as percentages with (95% confidence intervals). *P*-values concern the comparison with QFR.

**Table 4:** Per vessel diagnostic performance in patients with and without MI on CMR

Vessels of patients without MI on CMR							
	QFR (*n* = 142)	SPECT (*n* = 138)	*P*-value	PET (*n* = 139)	*P*-value	CMR (*n* = 135)^a^	*P*-value
Sensitivity	72 (54–86)	36 (21–55)	0.004	47 (29–65)	0.014	30 (16–50)	<0.001
Specificity	88 (80–93)	75 (66–82)	0.019	78 (70–85)	0.064	81 (72–88)	0.124
NPV	93 (86–96)	82 (73–88)	0.003	86 (78–92)	0.040	82 (73–88)	0.001
PPV	60 (43–75)	26 (15–42)	<0.001	35 (22–52)	0.003	28 (14–46)	0.001
Accuracy	85 (78–90)	67 (58–74)	0.001	72 (64–79)	0.006	70 (62–77)	0.002

Vessels of patients with MI on CMR							
	QFR (*n* = 186)	SPECT (*n* = 181)	*P*-value	PET (*n* = 184)	*P*-value	CMR (*n* = 171)^a^	*P*-value
Sensitivity	72 (57–83)	52 (37–66)	0.038	65 (50–78)	0.402	35 (22–51)	<0.001
Specificity	87 (81–92)	68 (60–75)	<0.001	57 (48–65)	<0.001	84 (77–89)	0.455
NPV	91 (85–95)	82 (74–88)	0.008	84 (75–90)	0.026	82 (74–87)	0.002
PPV	63 (49–75)	33 (23–45)	<0.001	31 (23–42)	<0.001	40 (25–57)	0.008
Accuracy	84 (78–88)	64 (57–71)	<0.001	59 (51–66)	<0.001	73 (66–79)	0.009

Values are presented as percentages with (95% confidence intervals). *P*-values concern the comparison with QFR.

^a^ Some patients underwent function and LGE CMR only and did not undergo perfusion CMR, as such the number of vesselswith a CMR MPI result is lower than that of QFR, SPECT, or PET despite patients being stratified based on the presence of MI on CMR.

## Discussion

This study compared the diagnostic performance of QFR with MPI in patients with prior MI and/or PCI. QFR exhibited a higher diagnostic accuracy and an AUC as compared with SPECT, PET, and CMR. Also when stratified according to the presence of MI on CMR, an invasive approach with functional evaluation by QFR yielded a higher accuracy and an AUC than assessment with MPI. These exploratory analyses suggest that in a population with prior CAD, a direct invasive strategy in conjunction with QFR results in a higher diagnostic certainty than non-invasive ischaemia detection when referenced by invasive FFR.

### The diagnostic performance of QFR and the influence of advanced CAD

In prospective studies, per vessel sensitivity and specificity of QFR ranged from 74 to 95% and from 86 to 92%, respectively.^8,14–16^ The sensitivity (74%) of QFR in the present study is in line with these findings and comparable with the sensitivity observed in the PACIFIC-I sub-study (72%).^17^ Nevertheless, sensitivity is at the lower range of reported percentages, which may be explained by the fact that QFR analyses were retrospectively performed. QFR analyses during ICA (online) presumably results in a better QFR and FFR concordance as pressure wire location and the distal marker in the QFR analyses can be directly matched; furthermore, the QFR analyst can interact with the operator in order to obtain optimal ICA images for QFR computation. In line with the above, the sensitivities of studies that utilized offline computation were 72, 74, and 77% against 87 and 95% for studies which obtained QFR online.^8,14–17^ Specificity (82%), on the other hand, is numerically lower than previously described which may be attributed to the studied population, i.e. patients with documented CAD.^17,18^ These patients have more advanced CAD as compared with the population included in the prospective QFR trials (26% had prior revascularization).^18^ As atherosclerosis is a progressive disease, patients with more advanced CAD are at an increased risk of suffering from both epicardial stenosis as well as from coronary microvascular dysfunction (CMD), which is an important contributor to QFR and FFR discordance.^19,20^ Mejia-Renteria *et al.*^19^ demonstrate that in patients with CMD (defined by a high index of microcirculatory resistance, IMR), QFR suffers from an increased rate of false positive findings hampering its specificity. This can be explained by the fact that FFR is directly influenced by microcirculatory resistance, whereas QFR does not determine the microvascular resistance but adjusts its boundary conditions based on lumen volume and contrast frame counting.^21,22^ However, contrast frame counting performed during QFR analysis does not correlated with IMR, which might explain the diminished performance of QFR in patients with CMD.^19^ Interestingly, the diagnostic performance of fixed and contrast QFR was similar among patients with CMD, as is also observed in the present study.^19^ Data regarding the performance of QFR in vessels with prior MI are scarce.^23^ In the study of Emori *et al.*,^23^ accuracy of QFR did not significantly differ between vessels with or without prior MI (87 vs. 92%). Similarly, we did not observe distinct differences in the performance of QFR among patients and vessels with or without MI on CMR.

### QFR vs. MPI

A direct invasive approach with functional evaluation of epicardial CAD and non-invasive ischaemia detection by means of MPI are both guideline-recommended diagnostic pathways for patients with a high pre-test probability of obstructive disease.^3^ There are, however, distinct differences between assessment of CAD by QFR/FFR or non-invasive MPI that should be considered when interpreting the present results.^12^ QFR and FFR solely assess the haemodynamic consequences of epicardial CAD, whereas MPI measures perfusion through the epicardial coronaries and microvasculature taking into account the effect of epicardial stenosis as well as CMD.^12,24^ Nonetheless, FFR is affected by the microvasculature and its resistance, e.g. patients with an epicardial stenosis and a healthy microvasculature may have an abnormal FFR but normal myocardial perfusion, whereas the same stenosis in patients with CMD may lead to a normal FFR but diminished myocardial perfusion.^12,22,25,26^ Notwithstanding the intricate interactions between the atherosclerotic process, epicardial flow, and myocardial perfusion, it is of clinical importance to evaluate the ability of diagnostic modalities to determine FFR-defined disease, as FFR-guided revascularization results in a lower rate of non-fatal MI and greater alleviation of symptoms as compared with MT.^1,2^ Similar to the PACIFIC-1 sub-study, QFR exhibited a higher diagnostic performance than non-invasive MPI.^17^ Given the above, it is not surprising that QFR and FFR, as measures of epicardial disease, correlate well with one another and that QFR has a good accuracy for determining FFR-defined significant CAD. Myocardial perfusion imaging by SPECT, PET, and CMR results in a higher rate of false negative findings (lower sensitivity) as compared with QFR which may be driven by several factors. First, an abnormal FFR does not necessarily commensurate with abnormal perfusion. Second, radiopharmaceuticals and contrast agent used in SPECT and CMR suffer from the roll-off phenomenon resulting in under-estimation of perfusion, which has a more pronounced impact with increasing MBF.^24^ Regarding specificity, SPECT and PET have a higher rate of false positive findings as compared with QFR. These false positive findings may be ascribed to the presence of CMD and myocardial scar in this high-risk population. Myocardial scar and CMD can result in diminished perfusion with a normal FFR (even in the absence of intermediate epicardial stenosis); furthermore, presence of scar can complicate the interpretation of MPI.^12,22^ In contrast, QFR and CMR have a similar rate of false positive findings. CMR may be less prone to false positive findings as compared with SPECT and PET, as it can accurately differentiate between myocardium and scar tissue.^27^ Despite the lower diagnostic accuracy of MPI, it should be noted that the MR-INFORM trial demonstrated that referral for ICA and subsequent revascularization after MPI by CMR resulted in a similar outcome as compared with a direct invasive approach with FFR.^28^ Nevertheless, when the goal is to determine the presence of FFR-defined significant CAD, ICA in conjunction with QFR leads to a higher diagnostic accuracy as compared with MPI.

### Limitations

The present study is limited by an overall QFR analysis success rate of 68%. This is driven by the fact that ICA images of 42% of the vessels eligible for QFR analysis were acquired without the use of a dedicated QFR acquisition protocol leading to a success rate of 52% among these vessels. The recently published FAVOR-III China provides insights into QFR analysis success rate using a fully optimized QFR acquisition protocol.^29^ The FAVOR-III randomized patients to a QFR or an angiography-guided revascularization strategy; of the 5881 patients assessed for eligibility, only 61 (1%) were excluded because of poor angiographic image quality and 70 (1.1%) were excluded because of severe vessel overlap or tortuosity. Furthermore, the QFR analysis success rate among vessels randomized to QFR-guided PCI was 99.9% (2725 of 2727 vessels).^29^ Although the impact of personalized segmentation seems to have little effect on the diagnostic performance of MPI, the present study used the standardized American Heart Association 17-segment model to assign segments to vascular territories for the MPI modalities, wherein differences between the standardized model and true anatomy cannot be ruled out.^10,30^ As QFR is based on ICA, possible discordances between a model and true anatomy have no effect on QFR. Furthermore, the present study excluded vascular territories in which significant CAD was solely defined by the presence of a sub-total or total occlusion. These vascular territories would largely be correctly assessed by MPI augmenting diagnostic performance (see Supplementary data online, *Table S8*). However, exclusion of these vessels did not influence a comparison of QFR and MPI as these vessels would also be correctly assessed by ICA in conjunction with QFR. It should be noted that the PACIFIC-2 study uses a hyperaemic MBF ≤2.3 mL/min/g to define FFR-defined significant CAD for all patients as there are no age- or risk-factor-dependent cut-offs. However, age influences hyperaemic MBF and the diagnostic performance of [^15^O]H_2_O PET, because such age- and/or risk factor-dependent cut-offs may improve the diagnostic performance of quantitative PET.^11^ Furthermore, the present study utilized a visual assessment of CMR, whereas diagnostic yield may increase when quantitative analyses are employed as demonstrated by recent studies.^31,32^ Lastly, the diagnostic performance is determined using a binary FFR result as reference standard. A QFR of 0.78 and a vascular hyperaemic MBF of 2.2 mL/min/g on PET with an FFR of 0.82 are considered false-positive findings; however, they might not reflect inaccuracies of the diagnostic modalities but rather are an expression of variability of measurements and utilization of binary cut-offs. This is substantiated by the higher performance of the diagnostic modalities in vessels with an FFR outside the grey zone (see Supplementary data online, *Table S7*).

## Conclusion

In patients with prior MI and/or PCI, QFR had a higher diagnostic performance than SPECT, PET, or CMR. Also in patients with MI on CMR, invasive assessment by QFR provided a greater diagnostic accuracy as compared with MPI. These exploratory analyses suggest that in patients with a high pre-test probability in which guidelines indicate that both an invasive approach and non-invasive ischaemia detection are feasible diagnostic strategies, a direct invasive approach utilizing QFR may yield a higher diagnostic certainty as compared with MPI when referenced by invasive FFR.

## Supplementary data

Supplementary data are available at *European Heart Journal - Cardiovascular Imaging* online.

## Supplementary Material

jead197_Supplementary_Data

## Data Availability

The data underlying this article will be shared on reasonable request to the corresponding author.
