# Supplementary material for: The diagnostic performance of quantitative flow ratio and perfusion imaging in patients with prior coronary artery disease
Source: Eur Heart J Cardiovasc Imaging. 2023 Aug 14;25(1):116–26. doi: 10.1093/ehjci/jead197 (PMC10735295; doi:10.1093/ehjci/jead197)
Supplement: jead197_Supplementary_Data [file jead197_supplementary_data.docx]

**Supplemental Information 1. Lesion and invasive coronary angiography characteristics prohibiting quantitative flow ratio analyses.**

Lesion characteristics:

-Culprit lesions of patients with an acute coronary syndrome.

-Bifurcation lesions with a Medina 1-1-1 classification.

-Aorta-ostial artery stenosis or ostial right coronary artery stenosis.

-Distal left main lesions in combination with a proximal circumflex lesion.

-Vessels with retrograde filling.

-Bypass grafts.

-Grafted coronary arteries.

-Myocardial bridging.

Invasive coronary angiography characteristics:

-Inadequate contrast injection leading to poor contrast opacification of the vessel.

-Too much overlap of other vessels with the lesion or areas just around the lesion in the target vessel in one or both of the angiographic acquisitions.

-Too much foreshortening of the target coronary artery in one or both angiographic acquisitions.

**Supplemental Figure 1. Correlation and agreement of contrast and fixed QFR with FFR.** Scatterplots with Spearman’s correlation coefficient and linear regression equations and Bland-Altman plots with ICC demonstrating the correlation and level of agreement of contrast and fixed QFR with FFR. Abbreviations: FFR: fractional flow reserve, ICC: intraclass correlation coefficient, SD: standard deviation, and QFR: quantitative flow ratio.

**
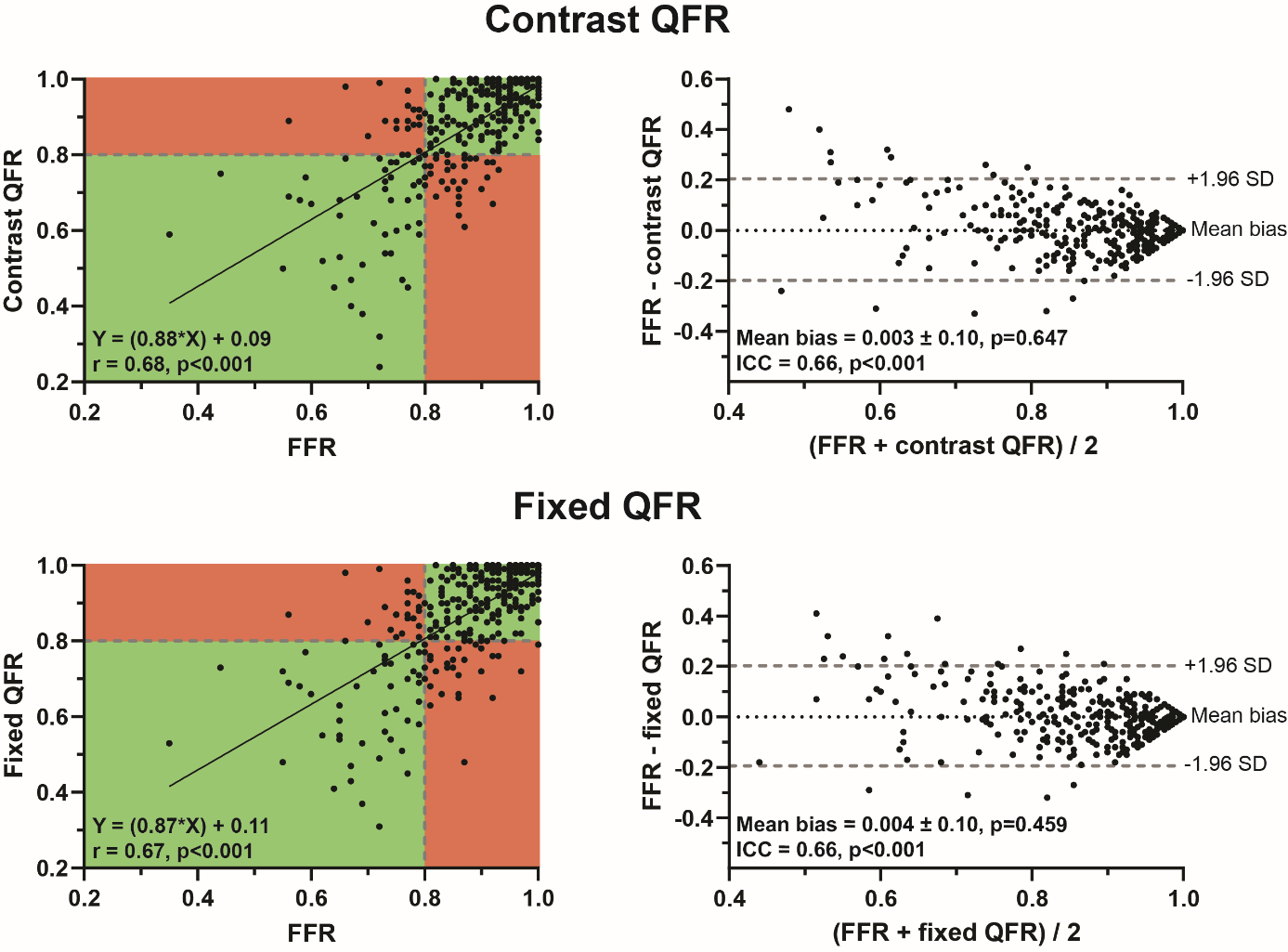
**

**Supplemental Figure 2. AUCs of contrast and fixed QFR for assessment of FFR defined significant CAD.** Comparative AUC analysis of contrast and fixed QFR. Abbreviations: area under the receiver operating characteristic curve, other abbreviations as in Supplemental Figure 1.

**
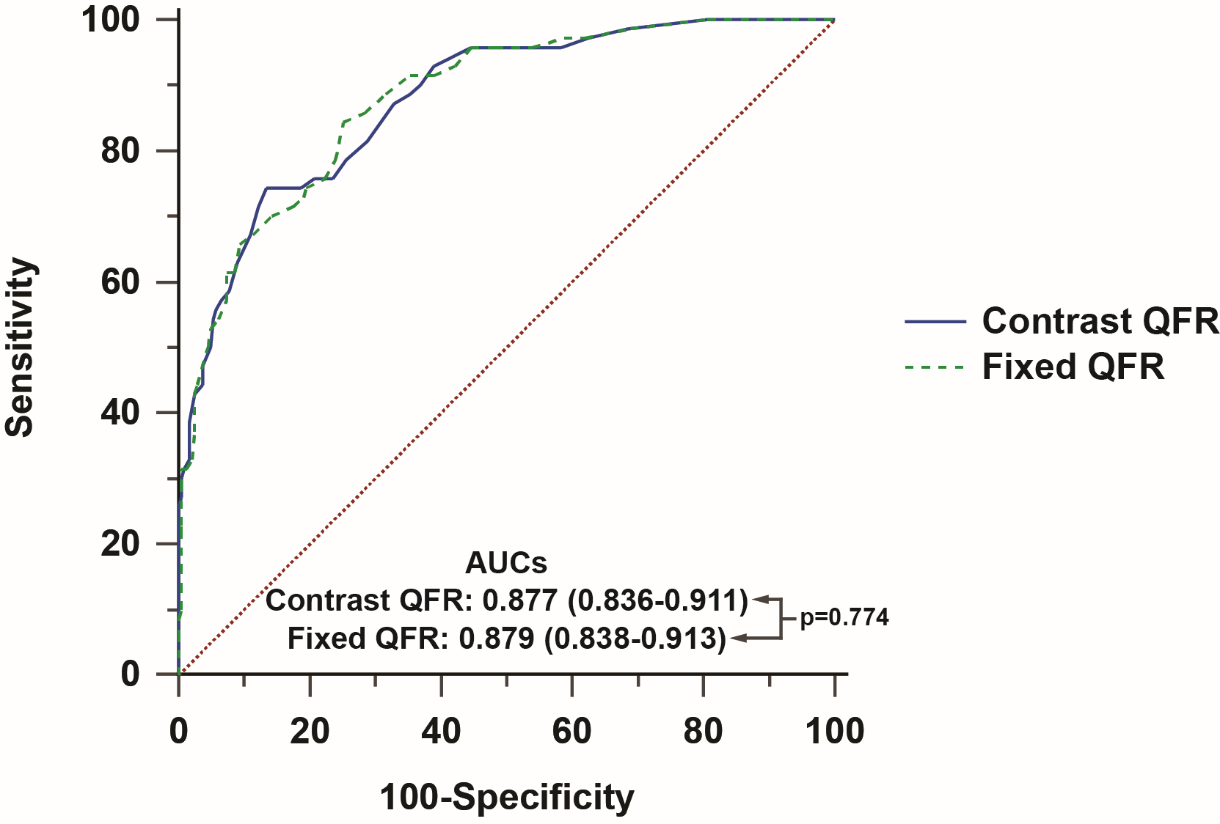
**

**Supplemental Figure 3. AUC of QFR and perfusion imaging in vessels without and with MI in the respective vascular territory on CMR.** AUC of QFR and comparative AUC analyses of QFR and perfusion imaging in vessels with the 4 modalities available, among vessels without MI in the respective vascular territory and with MI in the respective vascular territory on CMR. P-values in the comparative AUC analyses concern the comparison with QFR. Abbreviations: CMR: cardiac magnetic resonance imaging, MI: myocardial infarction, PET: positron emission tomography, SPECT: single-photon emission computed tomography, other abbreviations as in Supplemental Figure 1 and 2.

**
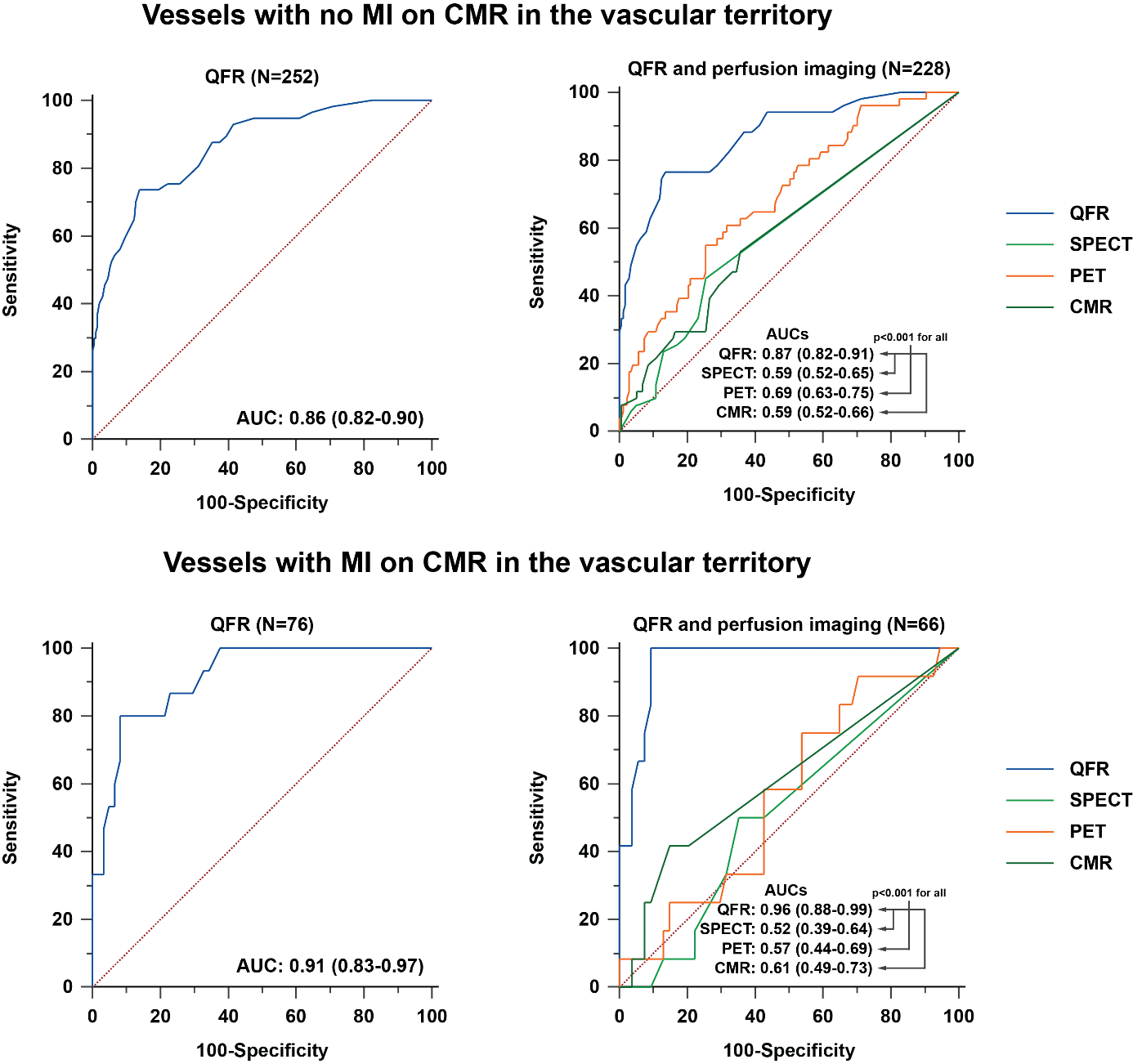
**

**Supplemental Table 1. Recommended ICA acquisition angles for QFR analyses.**

| **Vessel/bifurcation** | **First angulation** | **Second angulation** |
| --- | --- | --- |
| **Left main + LAD/Cx** | RAO 20, CAU 45 | AP, CAU 10 |
| **LAD/diagonal** | AP, CRA 45 | RAO 30, CRA 20 |
| **Cx/OM** | LAO 10, CAU 25 | RAO 25, CAU 25 |
| **RCA** | LAO 45, CAU 10 | LAO 20, CRA 20 |

Abbreviations: AP: anterior-posterior, CAU: caudal, CRA: cranial, Cx: circumflex artery, ICA: invasive coronary angiography, LAD: left anterior descending artery, LAO: left anterior oblique, OM: obtuse marginal, QFR: quantitative flow ratio, RAO: right anterior oblique, and RCA: right coronary artery.

**Supplemental table 2. Patient characteristics of in- and excluded patients.**

| **Patient characteristics** | **Included patients (N=166)** | **Excluded patients (N=23)** |
| --- | --- | --- |
| Male gender | 130 (78) | 23 (100) |
| Age in years | 63.1 ± 9.3 | 64.6 ± 9.4 |
| Body mass index (kg/m^2^) | 27.3 ± 4.2 | 28.5 ± 3.0 |
| **Cardiovascular risk factors** |  |  |
| Diabetes Mellitus | 35 (21) | 4 (17) |
| Hypertension | 105 (63) | 17 (74) |
| Hypercholesterolemia | 113 (68) | 15 (65) |
| Current smoker | 22 (13) | 4 (17) |
| History of smoking | 71 (43) | 7 (30) |
| Family history of CAD | 86 (52) | 9 (39) |
| **Medication** |  |  |
| Single antiplatelet therapy | 106 (64) | 14 (60) |
| Dual antiplatelet therapy | 59 (36) | 6 (35) |
| Beta-blocker | 102 (61) | 13 (57) |
| Calcium channel blocker | 59 (36) | 6 (26) |
| Statin | 142 (86) | 21 (91) |
| Long acting nitrate | 38 (23) | 12 (52) |
| ACE-inhibitor or AR-blocker | 95 (57) | 16 (70) |
| **Cardiac history** |  |  |
| Previous percutaneous coronary intervention | 157 (93) | 17 (74) |
| Previous myocardial infarction | 87 (52) | 13 (57) |
| **Symptoms** |  |  |
| Typical angina pectoris | 64 (39) | 14 (61) |
| Atypical angina pectoris | 41 (25) | 3 (13) |
| Non-specific chest pain | 25 (15) | 3 (13) |
| Dyspnea | 36 (22) | 3 (13) |
| **Left ventricular function** |  |  |
| LVEF% ^a^ | 58.7 ± 8.5 | 56.0 ± 11.3 |
| ≥55% | 124 (75) | 15 (65) |
| 45-<55% | 30 (18) | 3 (13) |
| 35-<45% | 9 (5) | 2 (9) |
| ≤35% | 3 (2) | 2 (9) |
| **Invasive coronary angiography** |  |  |
| Significant CAD | 100 (60) | 19 (83) |

Values are presented as; mean ± SD, median [interquartile range], or absolute numbers (%).

^a^ LVEF as measured on CMR. SPECT resting LVEF was used for three patients without CMR.

Abbreviations: ACE: angiotensin-converting enzyme, AR: angiotensin receptor, CAD: coronary artery disease, CMR: cardiac magnetic resonance imaging, LVEF: left ventricular ejection fraction, SPECT: single-photon emission computed tomography.

**Supplemental table 3. Angiographic, physiological, and imaging characteristics of in- and excluded vessels.**

|  | **Included vessels** | **Excluded vessels** |
| --- | --- | --- |
| **Vascular territory** | **N=334** | **N=233** |
| Right coronary artery | 93 (28) | 96 (41) |
| Left anterior descending artery | 127 (38) | 62 (27) |
| Circumflex artery | 114 (34) | 75 (32) |
| **Anatomical lesion characteristics^a^** |  |  |
| Lesion length (mm) | 16.3 [10.5-26.2] | - |
| Diameter stenosis (%) | 41 ± 14 | - |
| Intermediate lesions | 258 (77) | - |
| Area stenosis (%) | 56 ± 18 | - |
| Minimal lumen diameter (mm) | 1.7 ± 0.5 | - |
| **Invasive physiology characteristics** |  |  |
| QFR | 0.92 [0.80-0.98] | - |
| QFR ≤0.80 | 85 (25) | - |
| FFR | 0.90 [0.82-0.96] | 0.90 [0.83-0.96]^b^ |
| FFR ≤0.80 | 72 (22) | 111 (48) |
| Gray zone FFR (≥0.75 and ≤0.85) | 78 (23) | 40 (17) |
| **PET** | **N=329** | **N=222** |
| Hyperemic MBF (ml/min/g) | 2.79 ± 0.98 | 2.39 ± 1.02 |
| Indicative of ischemia | 127 (38) | 121 (55) |
| **SPECT** | **N=325** | **N=227** |
| Summed difference score | 0 [0-1] | 0 [0-3] |
| Perfusion defect percentage | 0 [0-5] | 0 [0-11] |
| Normal scan | 162 (51) | 80 (34) |
| Fixed perfusion defect | 57 (18) | 39 (17) |
| Reversible perfusion defect | 55 (17) | 53 (23) |
| Mixed perfusion defect | 51 (16) | 55 (24) |
| Indicative of ischemia | 106 (33) | 108 (48) |
| **CMR** | **LGE: N=328 Perfusion: N=306** | **LGE: N=221 Perfusion: N=198** |
| LGE score | 0 [0-1] | 0 [0-3] |
| Myocardial infarction | 76 (23) | 78 (34) |
| Perfusion defect score | 0 [0-3] | 1 [0-5] |
| Perfusion defect percentage | 0 [0-13] | 5 [0-25] |
| Indicative of ischemia | 64 (21) | 68 (34) |

Values are presented as; mean ± SD, median [interquartile range], or absolute numbers (%).

^a^Anatomical lesion characteristics are based on 3D quantitative coronary angiography data derived from QFR analysis and as such is not available in the excluded vessels.

^b^This concerns the median FFR of measured FFR. Subtotal or total occlusions were not classified as having FFR (e.g. a standard FFR of 0.5 for subtotal or total occlusion), therefore median FFR is similar between in- and excluded vessels but significant CAD as defined by FFR is more prevalent in the excluded vessels.

Abbreviations: FFR: fractional flow reserve, PET: positron emission tomography, MBF: myocardial blood flow, LGE: late gadolinium enhancement, other abbreviations as in Supplemental table 1 and 2.

**Supplemental Table 4. QFR analysis success rate and reasons QFR could not be computed stratified for vessel acquired without and with a standardized QFR ICA acquisition protocol.**

|  | **Vessel without QFR acquisition protocol (N=203)** | **Vessel with QFR acquisition protocol (N=285)** | **p-value** |
| --- | --- | --- | --- |
| **QFR analysis successful** | 104 (52) | 230 (81) | <0.001 |
| **Vessel characteristics preventing QFR analysis** | 2 (1) | 13 (5) | <0.001 |
| **ICA images preventing QFR analysis** | 97 (48) | 42 (15) | <0.001 |
| **Reasons QFR not performed** | **N=99** | **N=55** |  |
| **Vessel characteristics preventing QFR analysis** | **2 (2)** | **13 (24)** |  |
| Myocardial bridging | 2 (2) | 4 (7) |  |
| Ostial RCA lesion | 0 (0) | 1 (2) |  |
| Left main lesions in combination with proximal Cx | 0 (0) | 1 (2) |  |
| Medina 1-1-1 bifurcation lesion | 0 (0) | 3 (6) |  |
| Severe tortuosity | 0 (0) | 3 (6) |  |
| No healthy reference | 0 (0) | 1 (2) |  |
| **ICA images preventing QFR analysis** | **97 (98)** | **42 (76)** |  |
| Foreshortening | 30 (31) | 11 (20) |  |
| Poor contrast opacification of the vessel | 17 (18) | 14 (26) |  |
| Vessel overlap | 8 (8) | 13 (24) |  |
| End-diastolic frames not 25° apart | 17 (18) | 2 (4) |  |
| End-diastolic frame out of image | 14 (14) | 2 (4) |  |
| Frame rate <12.5 frames per second | 5 (5) | 0 (0) |  |
| Panning during filming | 6 (6) | 0 (0) |  |

Values are presented absolute numbers (%).

Abbreviations as in supplemental Table 1.

**Supplemental Table 5. Diagnostic performance of contrast and fixed QFR.**

|  | **Contrast QFR (N=317)** | **Fixed QFR (N=334)** | **p-value** |
| --- | --- | --- | --- |
| **Sensitivity** | 72 (61-81) | 68 (56-78) | 0.083 |
| **Specificity** | 87 (83-91) | 88 (84-92) | 0.618 |
| **NPV** | 92 (87-94) | 91 (87-94) | 0.348 |
| **PPV** | 63 (51-72) | 61 (50-71) | 0.694 |
| **Accuracy** | 84 (80-88) | 84 (79-88) | 0.787 |

Values are presented as percentages with (95% confidence intervals).

Abbreviations; NPV: negative predictive value, PPV: positive predictive value, other abbreviations as in Supplemental Table 1.

**Supplemental Table 6. Per vessel diagnostic performance of QFR, SPECT, PET, and CMR in vessels without and with myocardial infarction in the respective vascular territory on CMR.**

| **Vessels without myocardial infarction on CMR in the vascular territory** | | | | | | | |
| --- | --- | --- | --- | --- | --- | --- | --- |
|  | **QFR (N=252)** | **SPECT (N=244)** | **p-value** | **PET (N=247)** | **p-value** | **CMR (N=237)** | **p-value** |
| **Sensitivity** | 70 (57-81) | 43 (31-57) | 0.002 | 56 (43-68) | 0.059 | 32 (21-45) | <0.001 |
| **Specificity** | 87 (82-91) | 75 (68-81) | 0.002 | 72 (65-78) | <0.001 | 82 (76-87) | 0.161 |
| **NPV** | 91 (86-94) | 82 (76-87) | 0.001 | 85 (79-90) | 0.019 | 80 (74-85) | <0.001 |
| **PPV** | 62 (49-73) | 33 (23-45) | <0.001 | 36 (27-47) | <0.001 | 34 (22-48) | <0.001 |
| **Accuracy** | 83 (78-87) | 68 (62-73) | <0.001 | 68 (62-74) | <0.001 | 71 (64-76) | <0.001 |
| **Vessels with myocardial infarction on CMR in the vascular territory** | | | | | | | |
|  | **QFR (N=76)** | **SPECT (N=73)** | **p-value** | **PET (N=76)** | **p-value** | **CMR (N=69)** | **p-value** |
| **Sensitivity** | 80 (53-93) | 53 (29-76) | 0.129 | 67 (41-85) | 0.301 | 38 (16-66) | 0.010 |
| **Specificity** | 89 (78-94) | 57 (44-69) | <0.001 | 49 (37-62) | <0.001 | 84 (73-92) | 0.488 |
| **NPV** | 95 (85-98) | 83 (68-91) | 0.040 | 86 (70-94) | 0.073 | 87 (76-94) | 0.152 |
| **PPV** | 63 (40-81) | 24 (13-42) | <0.001 | 24 (14-40) | <0.001 | 36 (16-52) | 0.032 |
| **Accuracy** | 87 (77-93) | 57 (45-67) | <0.001 | 53 (41-64) | <0.001 | 76 (65-85) | 0.096 |

Values are presented as percentages with (95% confidence intervals).

P-values concern the comparison with QFR.

Abbreviations as in Supplemental Table 1, 2 and 3.

**Supplemental Table 7. Per vessel diagnostic performance of QFR, SPECT, PET, and CMR in vessel with an FFR <0.75 or >0.85.**

|  | **QFR (N=256)** | **SPECT (N=249)** | **p-value** | **PET (N=252)** | **p-value** | **CMR (N=237)** | **p-value** |
| --- | --- | --- | --- | --- | --- | --- | --- |
| **Sensitivity** | 87 (72-94) | 57 (41-72) | 0.005 | 75 (59-86) | 0.147 | 44 (29-61) | <0.001 |
| **Specificity** | 91 (86-94) | 70 (64-76) | <0.001 | 69 (62-75) | <0.001 | 81 (75-86) | 0.003 |
| **NPV** | 98 (94-99) | 90 (85-94) | 0.003 | 94 (89-97) | 0.074 | 89 (84-93) | <0.001 |
| **PPV** | 62 (49-74) | 25 (17-35) | <0.001 | 29 (21-39) | <0.001 | 30 (19-43) | <0.001 |
| **Accuracy** | 90 (86-93) | 68 (62-74) | <0.001 | 70 (64-75) | <0.001 | 76 (70-81) | <0.001 |

Values are presented as percentages with (95% confidence intervals).

P-values concern the comparison with QFR.

Abbreviations as in Supplemental Table 1, 2, and 3.

**Supplemental Table 8. Per vessel diagnostic performance of SPECT, PET and CMR among in- and excluded vessels.**

|  | **Included vessels** | **Excluded vessels** |
| --- | --- | --- |
|  | **SPECT** | |
| **Sensitivity** | 46 (34-57) | 68 (59-76) |
| **Specificity** | 71 (65-76) | 71 (61-78) |
| **NPV** | 83 (77-87) | 70 (61-78) |
| **PPV** | 30 (22-40) | 69 (59-77) |
| **Accuracy** | 66 (60-71) | 69 (63-75) |
|  | **PET** | |
| **Sensitivity** | 58 (46-69) | 82 (74-88) |
| **Specificity** | 67 (61-72) | 73 (64-81) |
| **NPV** | 86 (80-90) | 80 (72-87) |
| **PPV** | 32 (25-41) | 75 (67-82) |
| **Accuracy** | 65 (60-70) | 78 (72-83) |
|  | **CMR** | |
| **Sensitivity** | 33 (23-45) | 51 (41-61) |
| **Specificity** | 83 (77-87) | 81 (72-88) |
| **NPV** | 82 (76-86) | 63 (54-71) |
| **PPV** | 34 (24-47) | 72 (60-81) |
| **Accuracy** | 72 (67-77) | 66 (60-73) |

Values are presented as percentages with (95% confidence intervals).

Abbreviations as in Supplemental Table 1, 2, and 3.
